# Fucoidan as a Potential Therapeutic for Major Blinding Diseases—A Hypothesis

**DOI:** 10.3390/md14020031

**Published:** 2016-02-03

**Authors:** Alexa Klettner

**Affiliations:** Department of Ophthalmology, University Medical Center, University of Kiel, 24105 Kiel, Germany; aklettner@auge.uni-kiel.de; Tel.: +0049-431-597-2401; Fax: +0049-431-597-3140

**Keywords:** fucoidan, age-related macular degeneration, diabetic retinopathy, oxidative stress, VEGF, complement

## Abstract

Fucoidan is a heterogeneous group of sulfated polysaccharide with a high content of l-fucose, which can be extracted from brown algae and marine invertebrates. It has many beneficial biological activities that make fucoidan an interesting candidate for therapeutic application in a variety of diseases. Age-related macular degeneration and diabetic retinopathy are major causes for vision loss and blindness in the industrialized countries and increasingly in the developing world. Some of the characteristics found in certain fucoidans, such as its anti-oxidant activity, complement inhibition or interaction with the Vascular Endothelial Growth factor, which would be of high interest for a potential application of fucoidan in age-related macular degeneration or diabetic retinopathy. However, the possible usage of fucoidan in ophthalmological diseases has received little attention so far. In this review, biological activities of fucoidan that could be of interest regarding these diseases will be discussed.

## 1. Introduction

Fucoidans are sulfated polysaccharides found in the cell-wall matrix of phaeophyceae and in some marine invertebrates that contain high amounts of l-fucose [1]. The structure of fucoidans is highly complex and may differ substantially in composition and chemical structure between the species, depending also on regional and seasonal influences, and even on the method of extraction [1,2,3]. Because of a wide variety of beneficial biological activities, fucoidans have been considered as a potential treatment option for different diseases; however, so far no therapeutic application has been developed [3]. A field in which the therapeutic options of fucoidan have received little attention so far is ophthalmology. In this review, the activities of fucoidan will be discussed that might indicate a beneficial effect for two major blinding diseases in the industrialized countries, diabetic retinopathy (DR) and age-related macular degeneration (AMD).

### 1.1. Basic Structure of the Posterior Part of the Eye

In order to obtain visual input from the surroundings, the photoreceptors of the retina need to be stimulated by light. These photoreceptors are maintained by a single-layered epithelium located beneath them, the retinal pigment epithelium (RPE). These cells have many functions in order to maintain vision, as for example they comprise the outer blood-retinal barrier, transport nutrients and waste, recycle the visual pigment, and secrete growth factors [4]. The supply of nutrients and oxygen for photoreceptors is mainly provided by the choroid, a bed of vessels located beneath the retina [5]. The transduction of the signal is conducted by the neuroretinal layers downstream of the photoreceptors, culminating in the ganglion cells that transduce the information via the optic nerve to the brain. These retinal layers also contain vessels to supply them with oxygen and nutrients. In order to protect the retina from danger, specific cells of the monocytic lineage can be found in the retina, the so-called microglia, which convey innate immunity protection [6]. Interestingly, primates including humans, in contrast to other mammals, have a specific area in their retina, called the macula. This region is anatomically specified to facilitate high acuity vision [7]. 

### 1.2. Age-Related Macular Degeneration

Age-related macular degeneration is the major cause of blindness and severe visual impairment in the industrialized countries [8]. While the early forms of AMD are generally asymptomatic, the two late forms of AMD are associated with a devastating loss of vision. In the late “dry” form of the disease, geographic atrophy, extensive areas of the macula display a degeneration of the photoreceptors and the RPE. In the exudative “wet” form of AMD, vessels grow from the choroid beneath and into the retina. These vessels are immature and leaky, inducing tissue-destructive effects on photoreceptors and RPE. In addition, fluid accumulation under the macula occurs. The pathogenesis of AMD is so far not completely elucidated, but several factors have been implicated to contribute [9,10]. Environmental factors such as smoking and genetic susceptibilities have been shown to be significantly associated with the development of AMD. In addition, oxidative stress [11], as well as inflammatory factors, has been implicated [9]. Here, the complement system is of highest interest, as most genetic susceptibilities are connected to the complement system—mainly with the alternative pathway of the complement system [12,13].

In addition, activation of the resident microglia of the retina is currently discussed as a potential pathogenic factor in AMD, though its contribution is not clear [6]. Similarly, the migration of macrophage in the subretinal space is discussed as a possible pathogenic factor, as a healthy retina is devoid of macrophages [14].

For the development of exudative AMD, the Vascular Endothelial Growth Factor (VEGF) has been identified as the most important factor and considered to be prerequisite for the development of choroidal neovascularizations (CNV) (see below). Anti-VEGF therapies are the current gold standard for wet AMD treatment [8]. However, these treatments hold no cure for the disease, and the inhibitors, injected intravitreally, have to be given on a regular base. Visual decline is common even under therapy [15]. New treatment options are clearly warranted.

### 1.3. Diabetic Retinopathy

Diabetes is a chronic disease that is characterized by hyperglycemia, either resulting from a deficiency of insulin secretion, e.g., by autoimmunogenic destruction of the insulin producing pancreatic cells, or by an impaired insulin action. Diabetic retinopathy (DR) is the most frequent complication of diabetes, and one of the leading causes of visual impairment and blindness in the working population, both in the developed and the developing world [16].

Early signs of DR include microaneurysms, the loss of pericytes from retinal capillaries, and a breakdown of the blood-retinal-barrier, as well as retinal hemorrhages and exudates [17,18]. The most important factor in developing DR is the blood glucose level. In fact, early glycemic control may have lasting beneficial effects [19]. Hyperglycemia induces oxidative stress and low grade inflammation. [17]. Microangiopathy due to hyperglycemia leads to vascular leakage, causing edema. Another important risk factor for DR is hypertension [20]. In diabetic retinopathy, retinal pericytes and endothelial cells may die of apoptosis; here, reactive oxygen species concomitant with NFκB activation have been described to be of importance [16]. Leukostasis, in which immune cells become trapped in the retinal capillaries, induce a leukocyte-endothelial reaction. This may lead to an upregulation of pro-inflammatory cytokines and also to an occlusion of retinal capillaries [16]. Macrophages and neutrophils seem to be important for these processes [21,22]. The lack of perfusion induces ischemia, which, in turn, activates hypoxia-inducible factor (HIF)-1α and, consequently, VEGF. This can lead to the uncontrolled growth of new vessels, inducing proliferative diabetic retinopathy (PDR). In addition, the alteration of the retina in diabetes can disrupt the barrier function of the retinal endothelial cells and lead to fluid accumulation in the macular region (macular edema) [16]. A key element in the development both of PDR and macular edema is the growth factor VEGF.

### 1.4. Vascular Endothelial Growth Factor

VEGF (used synonymously for VEGF-A) is the most important angiogenic factor in development and disease [23,24]. The loss of one allele of its gene is embryonically lethal, and VEGF has been implicated in the development of cancer vascularization and of choroidal and retinal neovascularizations [25,26]. Of note, VEGF is also active in the healthy retina, where it exerts protective effects on neurons and the RPE, and where it maintains the endothelium of the choriocapillaris [27].

VEGF is expressed in different isoforms due to alternative splicing of the VEGF gene [28]. A wide variety of VEGF isoforms has been described; however, the most important are VEGF165 and VEGF121 [29,30]. The isoforms differ in their molecular weight and their ability to bind heparin-like molecules [31].

In order to regulate angiogenesis, different isoforms of VEGF can bind to its receptors, the tyrosine kinases VEGF-receptor (VEGFR-)1 and VEGFR-2, and, depending on the isoform, neuropilins (NRP1, NRP2) as co-receptors [32,33]. Several downstream pathways are involved in VEGF signaling, such as the mitogen activated protein kinase (MAPK) ERK1/2 in mitogenic signaling, phosphatidyl-inositol 3 kinase (PI3K) and Akt in survival signaling, and endothelial nitric oxide synthase (NOS) and p38 in permeability increase [34,35].

VEGF is regulated by a plethora of factors and inducible by many stimuli [32]. The major stimulus for VEGF upregulation is hypoxia, and the major transcription factor conveying this induction is HIF-1α [36]. Other transcription factors of importance for VEGF regulation include Sp1, Stat3 and NFκB [37,38]. Sp-1 and NFκB have been shown to be of particular importance in constitutive VEGF expression in the RPE [39,40]. A stimulus of high importance for VEGF expression, also considered a major factor in AMD and DR development [41,42], is oxidative stress. The retina is a location of high oxidative stress [11], and the oxidative burden accumulates over a lifetime. VEGF regulation of oxidative stress differs from constitutive VEGF expression. For oxidative stress, a role of the MAPK ERK1/2 has been described that was not found for constitutive VEGF expression [43].

## 2. Fucoidan

### 2.1. Fucoidan and VEGF

Fucoidan displayed several anti-VEGF functions in a variety of systems and seems to interfere with VEGF-induced signaling. Studies have shown that fucoidan can downregulate HIF-1α/VEGF signaling under hypoxia, as well as downregulate the phosphorylation of PI3K/Akt signaling [44,45,46]. Fucoidan has been shown to reduce the expression of the VEGF-receptors and even more of the VEGF co-receptors neuropilin [47]. In this setting, fucoidan inhibited the binding of VEGF to human umbilical vein endothelial cells (HUVEC) and inhibited HUVEC proliferation in response to VEGF [47]. This effect can be enhanced by oversulfation of fucoidan (*Fucus vesiculosus*) [48]. The authors show that both normal and oversulfated fucoidan with a molecular weight of 100–130 kDa prevent VEGF-induced phosphorylation of VEGFR-2, most likely by preventing binding of VEGF to its target [48]. Fucoidan from the same source (*Fucus vesiculosus*) reduced the expression of VEGF in tumor-bearing mice, concomitantly with a decrease in NFκB expression [49], a transcription factor shown to be important for VEGF expression [37,40]. Fucoidan from *Undaria pinnatifida* significantly reduced the expression of VEGF in HUVEC, suppressing its angiogenic activity [50]. Interestingly, while generally only an effect on VEGF165 is shown, as fucoidan does not bind to VEGF121, in this study, a reduction of expression is found also for VEGF121 [50]. Autoregulatory pathways involving VEGF binding to the VEGFR-2 have been described in VEGF regulation, with a positive feedback loop being induced by VEGF binding to its VEGFR-2 [39], which may offer an explanation as to how VEGF121 can be affected by an agent that does not bind to VEGF121. Cancer cells, such as HeLa, also show a reduced expression and secretion of VEGF under fucoidan treatment [51].

In a study of Chen *et al.*, low molecular weight (LMW) fucoidan reduced tube-like structure formation in HUVEC in hypoxic but not in normoxic conditions [44], which, considering the physiological role of VEGF in the retina, would be a big benefit compared to anti-VEGF molecules applied today, which do not discriminate between physiological and pathological VEGF.

It is important to note that the actions of fucoidan on VEGF are dependent on the molecular weight, the degree of sulfation, the source of the fucoidan and its concentration, and that pro-angiogenic activities of fucoidan have also been described [52]. For example, LMW fucoidan extracted from *Fucus vesiculosus* has been shown to induce endothelial cell migration by enhancing the binding of VEGF165 to its receptors VEGFR-2 and NRP1, which seemed to be a dose-dependent effect, as tube formation by HUVEC was increased at concentrations of 1 µg/mL and 10 µg/mL, while at 100 µg/mL the effect became inhibitory [53]. Indeed, fucoidan is used in scaffolds to load them with VEGF165 and to intensify the vascularization response [54].

It also must be noted that most of these studies were conducted in HUVEC cells or tumor-bearing mice. For the ophthalmological setting, neither of these models is appropriate to indicate beneficial effects. In the ophthalmological setting, VEGF expression and HIF-1α induction were reduced in retinas of diabetic mice treated with LMW fucoidan from *Laminaria japonica* [45]. VEGF is also reduced in a dose-dependent manner in brain microvascular endothelial cells treated with LMW fucoidan from *Laminaria japonica* [45]. Moreover, a study conducted with fucoidan from *Fucus vesiculosus* in different retinal pigment epithelium model systems clearly showed a reduction of VEGF secretion and expression over time [55]. Interestingly, a reduction of expression can be found even when coapplied with the anti-VEGF reagent bevacizumab. Moreover, the angiogenic potential induced by VEGF165 or RPE supernatant was reduced by fucoidan in this setting, clearly indicating a potential beneficial effect in choroidal neovascularizations [55].

These data implicate a possible potential effect of fucoidans in ophthalmology to treat VEGF induced pathologies; however, the bioactivity and concentration of the respective fucoidan used must be meticulously tested in order to eliminate potential unwanted adverse effects ameliorating VEGF-induced conditions.

### 2.2. Fucoidan and Oxidative Stress

An important feature of diabetic retinopathy and in age-related macular degeneration is the prevalence of oxidative stress [16,56]. Oxidative stress can be induced by the exposure to high energy wavelength of the visible light spectrum, especially combined with the high oxygen tension found in the retina [11]. In addition, hyperglycemia as encountered in diabetes can induce oxidative stress in the tissue, e.g., by upregulating the generation of superoxide radicals [57].

Fucoidan has been shown to have anti-oxidative properties [58], predominantly by scavenging superoxide radicals [59]. The antioxidative ability varies between fucoidans of different sources, with a positive correlation between sulfate content and radical scavenging ability [59,60,61]. The exact mechanisms of the scavenging abilities have not been elucidated so far, but have been suggested to be related to molecular weight (as small polysaccharides provide more reducing ends) and to uronic acid content [60]. In addition, fucoidan has also been shown to induce the expression of the transcription factor nuclear factor erythroid-2 related factor 2 (Nrf2) [61], an important transcription factor in anti-oxidant defense in the RPE [11], and its target gene superoxide dismutase [62]. Reduced Nrf2 activity may be involved in the development of AMD, as Nrf2 knock-out mice develop an AMD-like pathology [63], and the knock-out of Nrf2 renders RPE cells highly susceptible to oxidative stress-induced cell death [64].

Its anti-oxidant and protective effects have been shown in several biological systems. In tumor cells, fucoidan of *Cladosiphon* decreased both intracellular and released hydrogen peroxide (H_2_O_2_) levels [51]. Concomitantly, fucoidan suppressed the secretion of VEGF (see above). LMW fucoidan from *Undaria pinnatifida* suppressed oxidative stress in RAW264.7 cells, a macrophage cell line [65], and fucoidan from *Ecklonia cava* protected from oxidative stress in a zebrafish model [66] or, obtained from *Fucus vesiculosus*, from oxidative stress in liver fibroses [62]. In addition, fucoidan from *Cladosiphon okamuranus* prevented the disruption of the intestinal barrier function induced by H_2_O_2_ in Caco-2 cells [67]. This might be of special interest in diabetic retinopathy, where increased paracellular permeability of the retinal endothelial cells contributes strongly to the development of diabetic macular edema [68].

Little is known on the antioxidant properties of fucoidans in cells of the eye, but fucoidan was shown to protect ARPE19 cells, a human RPE cell line, from oxidative stress-induced by high glucose [69]. It protected the cells from cell death and normalized the generation of reactive oxygen species. In addition, it inhibited the activation of ERK1/2, a major factor in oxidative stress-induced VEGF upregulation [43,69]. These data indicate that the anti-oxidative properties of fucoidan may be beneficial in AMD or diabetic retinopathy.

### 2.3. Fucoidan and Complement

The complement system is an enzyme cascade of the innate immune system that protects the organism from harm by facilitating phagocytic uptake, activation of immune cells and lysis of harmful cells or microorganisms, and it links the innate immunity with adaptive defense. The complement system can be activated mainly via the classical pathway, induced by the binding of the factor C1q to microorganisms or antibody/antigen complexes, or the alternative pathway, induced mainly by a spontaneous activation of the factor C3 [13].

Fucoidan has been described to inhibit the activation of the classical, and, to a lesser extent, of the alternative pathway. Fucoidan has been shown to bind to C1q and C4, inhibiting the first steps of the classical pathway activation [70]. Additionally, a partial effect on the activation of C3 was observed, suppressing the C3 convertase [70,71]. Of note, the binding of CFB to C3b can be inhibited by fucoidan [70], which may be of particular interest since polymorphism of the CFB gene may contribute to a higher risk for AMD [72].

The action of fucoidan on the complement system is strongly dependent on its molecular composition. Sulfate groups have been described to be necessary but not sufficient for anti-complement activity, and the anti-complement activity to be dependent on the content of galactose and glucuronic acid, as shown for fucoidans of *Ascophyllum nodosum* [73]. In addition, molecular weight is an important factor, yet no simple correlation of weight and anti-complement activity can be postulated, with different molecular weight showing an optimum of inhibition concerning the classical and the alternative pathway [73]. The same authors show that the inhibitory effect on the formation of the C3 convertase cannot be shown for fucoidans with MW below 16,600 kDa [71].

These data indicate a potential beneficial effect of fucoidan on the pathogenesis of AMD. However, it must be noted that the effect of fucoidan is mainly on the pathway of classical activation, while it is the alternative pathway that is implicated for AMD pathogenesis. Moreover, the direct role of the complement system on the development of AMD has not been elucidated so far. In addition, as described above, the effect of fucoidan on the complement system is highly dependent on their chemical composition and needs to be elucidated thoroughly before preclinical or clinical testing can be considered.

### 2.4. Fucoidan and Monocyte-Likes Cells (Microglia, Macrophages)

Microglia activation in the retina has been discussed as a factor for the development of AMD [6]. The role of macrophages in the pathology of AMD is under debate and may depend on their polarization [14,74,75]. In DR, the involvement of microglia on the development of diabetic retinopathy is under debate [76], while macrophages are likely to be involved in its pathogenesis [21,22].

For activated microglia from the brain, it has been shown that fucoidan is able to reduce the activation following stimulation with lipopolysaccharide (LPS). In particular, fucoidan from *Fucus vesiculosus* reduced the activation of NFκB and the MAPK JNK, ERK1/2 and p38, as well as the expression of iNOS, Cox2 and the monocyte-chemoattractant factor MCP-1 [77]. Similarly, on macrophages activated with LPS, fucoidan from *Ecklonia cava* downregulated the expression of iNOS, Cox2, TNFα and IL-1β [78]. Conversely, however, fucoidans isolated from *Laminaria angustata* activated macrophages, inducing TNFα and IL-6 production [79]. Similar results were obtained with fucoidan isolated from *Agarum cribosum,* with activated macrophages expressing iNOS, Cox-2 and IL-10 [80]. This shows that the effect of fucoidans on monocyte-like cells is not uniform and depends on cell type and fucoidan origin. This needs to be considered regarding a possible use of fucoidan for AMD or diabetic retinopathy, as induction of pro-inflammatory macrophage activation is clearly not desirable in these diseases.

### 2.5. Fucoidan and Diabetic Retinopathy

Fucoidan from several different sources has been shown to reduce the blood-glucose levels in different models of diabetes. In insulin-resistant mice, fucoidan from *Cucumaria frondosa* increased the mRNA expression of the insulin receptor, as well as insulin receptor substrate 1, PI3K/Akt and Glut4, a glucose transporter protein [46]. Additionally, it reduced the weight of diabetic mice, reduced blood glucose and enhanced insulin sensitivity. In diabetic rats, in which diabetes was induced by alloxan, fucoidan from *Saccarina japonica* reduced blood glucose levels, which was accompanied by increased serum insulin levels and altered plasma lipid levels [81]. In another study, fucoidan from *Undaria pinnatifida* was used in three different fractions of different molecular weight. All three fractions suppressed blood glucose in db/db mice, a diabetic mouse model, and improved insulin sensitivity, depending on their sulfate content [82]. In addition, it has been shown that fucoidan from *Sargassum wightii* inhibited the enzyme alpha-d glucosidase, which is important for the provision of glucose into the blood stream [83]. However, this feature seems to be highly dependent on the source of fucoidan, since, e.g., fucoidans from *Fucus vesiculosus* were far less potent than fucoidans from *Ascophyllum nodosum* [84]. First line studies in obese but non-diabetic humans found that orally administered fucoidan elevated the insulin level in these patients; however, an increase in insulin resistance was noted [85]. Taken together, these results indicate that fucoidan could help regulating the blood glucose level in a non-toxic way and therefore help to prevent the onset of DR in diabetic patients.

Apart from its influence on blood glucose levels, fucoidan has been shown to attenuate diabetic retinopathy in the mouse model [45]. In this study, LMW fucoidan from *Laminaria japonica* reduces retinal damage and retinal neovascularization, most likely via inhibiting HIF-1α activation of VEGF (for more information on the influence of fucoidan on VEGF please see above). LMW fucoidan also inhibited the high-glucose induced proliferation of microvascular cells [45].

As described above, hypertension is a strong risk factor for developing and exacerbating diabetic retinopathy. In a model using Goto-Kakizaki rats, LMW fucoidan from *Laminaria japonica* could be found to ameliorate hypertension in these rats and protect the endothelium by inducing eNOS activity and NO production [86]. Indeed, a similar observation could be made in obese human patients, in which a daily oral supplementation of 500 mg/mL fucoidan reduced diastolic blood pressure [85].

Low grade inflammation has been implicated in the development of diabetic retinopathy [87], with inflammatory cytokines such as TNFα, IL-1β, or inflammatory mediators such as iNOS found in the aqueous humor or epiretinal membranes. In addition to the above-mentioned effects of fucoidan on microglia and macrophages, fucoidan has shown anti-inflammatory properties. In an ischemia-reperfusion model, fucoidan from *Laminaria japonica* reduced the levels of TNF-α [88], while, in C6 glioma cells treated with TNFα, fucoidan from *Fucus vesiculosus* has been shown to suppress the expression of iNOS [89]. However, similar to what has been described for macrophages, fucoidan from *Undaria pinnatifilda* activates neutrophils, leading to a pro-inflammatory TNF-α [90]. Again, the effect of fucoidan on inflammatory parameters is dependent on the source of fucoidan and the cell- and injury models used.

## 3. Future Directions

Taken together, these data give much indication for a potential beneficial effect of fucoidans on age-related macular degeneration and diabetic retinopathy. Yet it must be noted that the effects of fucoidan on the different features important for either AMD or DR are strongly dependent on molecular characteristics, extraction methods, and the source of the fucoidan. Furthermore, the use of unsuited fucoidans could have undesired, possibly even aggravating effects. Therefore, pre-clinical testing should be done to develop a database of different fucoidan fractions in order to identify those that combine various beneficial effects for the respective disease. In a second step, the most promising fucoidans should be tested in relevant *in vitro* systems, and, finally, these fucoidans should be tested in the appropriate animal models, such as streptozotocin-induced diabetes in mouse for diabetic retinopathy and Nrf2 knock-out mice in age-related macular degeneration. These data can then pave the way for clinical phase-one studies.

## 4. Conclusions

**Figure 1:**
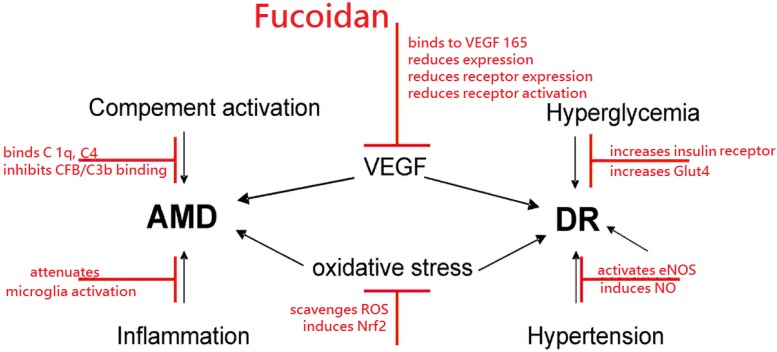
Schematic of potential beneficial effects of fucoidan, depicted in red, on age related macular degeneration (AMD) or diabetic retinopathy (DR). Additional abbreviations: complement component (C), complement factor B (CFB), endothelial nitric oxide synthase (eNOS), glucose transporter type 4 (Glut4), nitric oxide (NO), nuclear factor erythroid-2 related factor 2 (Nrf2), reactive oxygen species (ROS), Vascular Endothelial Growth Factor (VEGF).

Fucoidans have many characteristics that render them interesting substances for the treatment of the major blinding diseases diabetic retinopathy and age-related macular degeneration. They can protect from oxidative stress, reduce VEGF activity, interfere with complement activation, have immune-modulating effects on microglia, reduce blood hyperglycemia, attenuate diabetic retinopathy in rodent models and ameliorate hypertension. A detailed list on the effects of fucoidan in the different experimental models can be found in Table 1 (*in vitro* models) and Table 2 (*in vivo* models). A schematic of the potential beneficial effects of fucoidan is depicted in Figure 1. However, most of the described features are highly dependent on the source, molecular weight, sulfation and even concentration. In order to investigate fucoidan further in this field, a thorough investigation of the bioactivity of the respective fucoidan fraction must be undertaken to identify the most promising candidate for preclinical and clinical testing and to avoid unwanted serious adverse events.

**Table 1:** Effect of different fucoidans in different cell culture models.

Cell Type	Disease Model	Concentration (µg)	Source	Effect	Ref.
HUVEC	Hypoxia	25–100/mL	*Sargassum hemiphyllum*	Reduced tube formation	[44]
HUVEC	VEGF165 application	8/mL 10/mL	*Fucus vesiculosus*	Blocks VEGF165 binding	[47] [48]
HUVEC	VEGF165 application	10/mL	*Fucus vesiculosus*	Reduces VEGFR-phosporylation	[48]
HUVEC	-	100, 200, 400/mL	*Undaria pinnatifida*	Reduces VEGF	[50]
T24 bladder cancer	Hypoxia	50, 100/mL	*Sargassum hemiphyllum*	Reduces VEGF	[44]
Microvascular endothelial cells	High glucose	12.5, 25, 50/mL	*Laminaria japonica*	Reduces VEGF	[45]
HeLa uterine carcinoma	-	10%, 20% extracts	*Cladosiphon novae-caledoniae kylin*	Reduces VEGF	[51]
Arpe19 RPE cell line	-	100/mL	*Fucus vesiculosus*	Reduces VEGF	[55]
Primary RPE cells	-	100/mL	*Fucus vesiculosus*	Reduces VEGF	[55]
Vero kidney fibroblasts	Oxidative stress	25–200/mL	*Ecklonia cava*	Scavenges ROS	[66]
Caco-2 intestinal epithelial	Oxidative stress	2.5/mL	*Cladosiphon okamuranus Tokida*	Protects barrier function	[67]
BV2 microglia	LPS stimulation	25–100/mL	*Fucus vesiculosus*	Reduces iNOS, Cox2, IL-1β, TNFα	[77]
C6 glioma cells	TNFα stimulation	50/mL	*Fucus vesiculosus*	Reduces iNOS	[89]
Neutrophils	-	10/mL	*Undaria pinnatifilda*	Induces TNFα	[90]
Raw 264.7 macrophages	LPS stimulation	12.5–100/mL	*Ecklonia cava*	Reduces iNOS, Cox-2, IL-1β, TNFα	[78]

**Table 2:** Effect of different fucoidans in different animal models.

Animal	Disease Model	Concentration (mg)	Source	Effect	Ref.
Nude mice (BALP/c)	Tumor growth	80, 160, 300/kg	*Sargassum hemiphyllum*	Reduces growth	[44]
C57BL/6 mice	Streptozotocin-induced diabetes	50, 100, 200/kg	*Laminaria japonica*	Reduces VEGF (retina)	[45]
C57BL/6J mice	Insulin resistance	80/kg	*Cucumaria frondosa*	Ameliorates insulin resistance	[46]
BALB/cAnNCr mice	Tumor angiogenesis assay	1/0.2 mL saline	*Fucus vesiculosus*	Reduces angiogenesis	[47]
C57BL/6J mice BALB/cAnNCr	VEGF Matrigel angiogenesis	1/0.2 mL saline	*Fucus vesiculosus*	Reduces angiogenesis	[47]
C57BL/6J mice	Lewis lung carcinoma cells inoculation	1, 3/mice	*Fucus vesiculosus*	Declines VEGF, MMP, NFκB	[49]
Zebrafish	Oxidative stress	100, 200 µg/mL	*Ecklonia cava*	Scavenges radicals	[66]
Sprague-Dawley rats	Liver fibrosis	100/kg	*Fucus vesiculosus*	Activates Nrf2	[62]
Sprague-Dawley rats	Ischemia-reperfusion injury	100, 200/kg	*Laminaria japonica*	Reduces TNFα, NFκB	[88]
Goto-Kakizaki rats	Diabetes	50, 100, 200/kg	*Laminaria japonica*	Reduces hypertension	[86]
Wistar rats	Alloxan-induced diabetes	50/kg	*Saccharina japonica*	Reduces blood glucose	[81]
C57BL/KSJ mice	Diabetes	200, 1200/kg	*Undaria pinnatifida*	Reduces blood glucose	[82]
Human	Obesity	500	*Laminaria japonica* and *Cystoseira canariensis*	Reduces hypertension	[85]
